# Butterfly eyespots exhibit unique patterns of open chromatin

**DOI:** 10.12688/f1000research.133789.1

**Published:** 2023-10-31

**Authors:** Suriya Narayanan Murugesan, Antónia Monteiro

**Affiliations:** 1 Biological Sciences, National University of Singapore, Singapore, 117558, Singapore

**Keywords:** Chromatin accessibility, ATAC-Seq, Butterfly eyespots, Notch, wound healing

## Abstract

**Background:** How the precise spatial regulation of genes is correlated with spatial variation in chromatin accessibilities is not yet clear. Previous studies that analysed chromatin from homogenates of whole-body parts of insects found little variation in chromatin accessibility across those parts, but single-cell studies of *Drosophila* brains showed extensive spatial variation in chromatin accessibility across that organ. In this work we studied the chromatin accessibility of butterfly wing tissue fated to differentiate distinct colors and patterns in pupal wings of *Bicyclus anynana.*

**Methods:** We dissected small eyespot and adjacent control tissues from 3h pupae and performed ATAC-Seq to identify the chromatin accessibility differences between different sections of the wings.

**Results:** We observed that three dissected wing regions showed unique chromatin accessibilities. Open chromatin regions specific to eyespot color patterns were highly enriched for binding motifs recognized by Suppressor of Hairless (Su(H)), Krüppel (Kr), Buttonhead (Btd) and Nubbin (Nub) transcription factors. Genes in the vicinity of the eyespot-specific open chromatin regions included those involved in wound healing and SMAD signal transduction pathways, previously proposed to be involved in eyespot development.

**Conclusions:** We conclude that eyespot and non-eyespot tissue samples taken from the same wing have distinct patterns of chromatin accessibility, possibly driven by the eyespot-restricted expression of potential pioneer factors, such as Kr.

## Introduction

Dynamic changes in chromatin opening and closing in a cell nucleus facilitates the binding of transcription factors and the expression of nearby genes. Chromatin is often opened via the binding of pioneer transcription factors, that start the process of unwinding DNA from its highly packed configuration around histones. This unwinding then exposes potential cis-regulatory elements (CREs) to other transcription factors (TFs). If these TFs are present in the cell, and if the newly opened regulatory sequences contain their respective target binding sequences, this second set of TFs, with the help of the pioneer factors, co-operatively bind DNA and begin the process of either activating or repressing nearby genes ( Balsalobre and Drouin 2022; Iwafuchi-Doi *et al.* 2016).

Studies examining the opening and closing dynamics of chromatin obtained from different tissues showed that most genes share the same chromatin profile at specific points in development, but have a different profile at different stages of development. For example, chromatin extracted at the same stage of development from legs, halteres, and wings of *Drosophila*, had the same chromatin profile, except for chromatin around tissue-specific selector genes ( *e.g. Ultrabithorax* ( *Ubx*), *vestigial*), that was uniquely open in that tissue ( McKay, Estella, and Mann 2009; McKay and Lieb 2013). Chromatin profiles, however, were distinct between larval and pupal stages ( van der Burg *et al.* 2019), suggesting that global developmental progression, driven by hormonal signals, rather than by tissue-specific patterns of gene expression, produced these distinct chromatin profiles.

Similarly, work on *Heliconius* and *Junonia* butterflies’ forewings and hindwings showed that except for the hindwing selector gene ( *Ubx*), all other genes shared the same chromatin profile between the two wing types ( van der Burg *et al.* 2019; Lewis *et al.* 2019; Lewis and Reed 2019). This was the case even for genes that were expressed only in a specific region of the *Heliconius* forewing, like *Optix* in the red band area, which showed no difference in its chromatin profile between fore and hindwings ( Lewis *et al.* 2019; Lewis and Reed 2019). These results suggested, again, that all tissues sampled at the same time in development had the same chromatin profile.

Recent work performed at higher resolution, however, contradicted these initial results. Chromatin profiles from small sections of *Drosophila* embryos had differences in chromatin accessibility, which were not identified when using whole embryos ( Bozek *et al.* 2019). Similarly, there were extensive differences in chromatin accessibilities of single cells in developing brains of *Drosophila* ( Janssens *et al.* 2022). These studies suggest, instead, that the similar chromatin profiles observed across tissues in earlier studies may be a result of averaging out distinct signals across many cells ( McKay and Lieb 2013).

Here we explored whether variation in chromatin accessibility can be associated with gene expression differences in a highly patterned tissue. We examined chromatin accessibility across the forewings of *Bicyclus anynana* butterflies, focusing on a region with eyespot patterns and two control regions next door. Eyespots consist of multiple concentric rings of color, and the central cells of these pattern elements appear to be reusing part of a limb gene regulatory network (GRN) for their differentiation ( Murugesan *et al.* 2022). Hundreds of genes are differentially expressed (DE) in eyespot centers compared to adjacent regions in the wings ( Murugesan *et al.* 2022; Özsu and Monteiro 2017). It is currently unclear, however, whether the eyespot DE genes display unique patterns of chromatin accessibility, compared to other genes. It is also unclear to what extent chromatin from eyespot regions, in general, differs in accessibility from chromatin from other wing regions. Pioneer factors, evenly expressed across the wing, might create the same chromatin profile across all wing cells, but the expression of unique TFs in the eyespot regions could lead to hundreds of DE genes in those regions. Alternatively, pioneer factors, expressed specifically in the eyespots, could produce eyespot-specific open chromatin regions that contribute to the differential regulation of genes in that region alone.

To investigate these alternate hypotheses, we performed fine dissections of eyespot and two flanking control wing tissues from 3 h old pupal forewings and conducted assays for transposase-accessible chromatin with Sequencing (ATAC-Seq) separately on each tissue type, and also for a complete wing, to identify genomic areas with open chromatin. We then identified differences in chromatin accessibility across these wing regions. Having found eyespot-specific regions of open-chromatin, we conducted a motif enrichment analysis for these regions to identify potential pioneer factors and/or specific TFs associated with eyespot development. Finally, we examined the ontology of genes that mapped to the vicinity of the uniquely open chromatin of eyespot tissues to identify their general identities.

## Results

### Open chromatin varies between eyespot and non-eyespot regions

**Figure 1.**
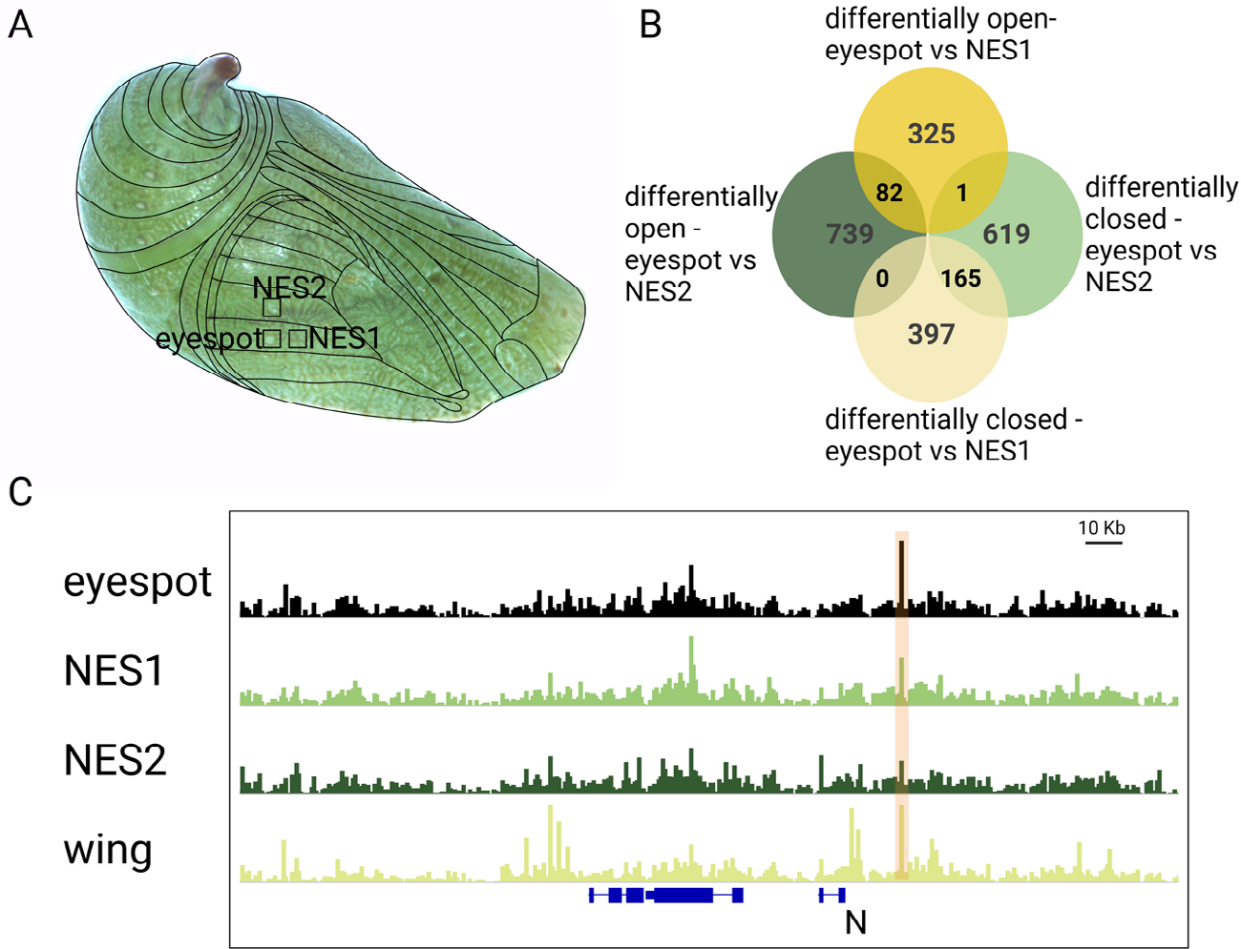
Differential chromatin accessibility between eyespots, control tissues, and whole wing. (A) Tissues chosen for the study were the eyespot region and two flanking control tissues (NES1 and NES2). (B) Number of peaks differentially open between different comparisons (differentially open- peaks are open in eyespot compared to NES1 or NES2 sector. Differentially closed – closed in eyespot and open in NES1 or NES2). (C) Example of an ATAC-peak next to the gene Notch that was DO in the eyespot region. Note how this peak was shown as open across the whole wing as well.

To identify region-specific open chromatin, we first identified ATAC-Seq peaks that were consistently present across biological replicates of each wing region, and then added them across wing regions. A total of 166,062 peaks were identified across the three wing regions. Differentially open (DO) peaks between eyespot and control regions were identified by pairwise comparisons, as described in Murugesan *et al.* (2022). A comparison of eyespots with a non-eyespot sector region (NES1) in a more proximal part of the wing identified 562 peaks (397 + 165) that were DO in NES1, and these 562 peaks mapped to the vicinity of 506 genes ( Figure 1B, Data S1). Similarly, a comparison of eyespots with a second non-eyespot sector region (NES2), in a more anterior part of the wing, identified 785 peaks (619 + 1 + 165) that were DO in NES2, and these peaks mapped to 647 genes ( Figure 1B, Data S1). A comparison of eyespots with the two non-eyespot sector regions (NES1 and NES2) identified 82 eyespot-specific DO peaks that mapped to 80 genes ( Figure 1B, 1C, Figure S1: Murugesan and Monteiro, 2023, Data S1). These data indicate that multiple distinct patterns of open chromatin are present across *B. anynana* 3 h old pupal forewings. Furthermore, only some of these patterns resemble the pattern of peaks obtained from ATAC-Seq performed on whole-wings of the same age ( Figure 1C, Data S1).

### Motif enrichment in open chromatin

**Figure 2.**
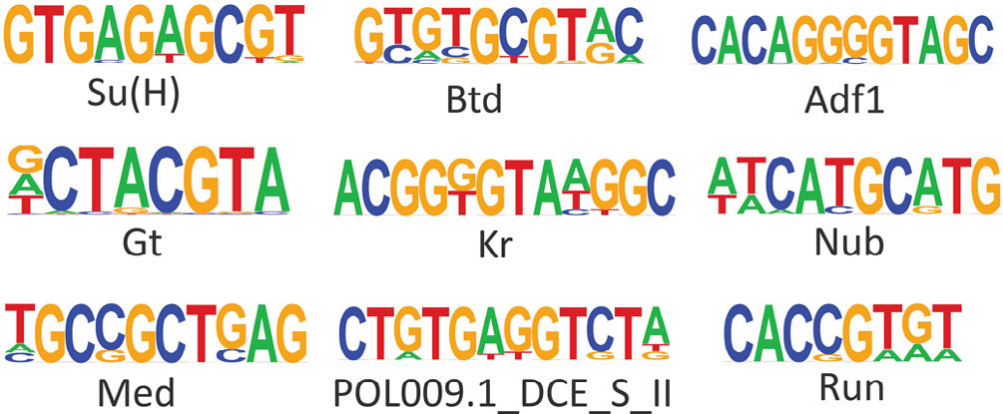
TF Motifs enriched for the eyespot-specific DO peaks. Suppressor of Hairless (Su(H)), Buttonhead (Btd), Adh transcription factor 1 (Adf1), Giant (Gt), Krüppel (Kr), Nubbin (Nub), Medea (Med), DCE_S_II predicted from JASPAR database, Runt (Run).

Open areas of chromatin often have specific genomic signatures/binding sites for pioneer TFs and for secondary TFs that have bound or will bind these regions in the future ( Galupa *et al.* 2023). To examine whether specific DNA motifs were enriched in DO peaks we performed motif enrichment analysis. We identified nine motifs that were enriched in the 82 eyespot-specific ATAC-Seq peaks. Binding site motifs for the transcriptional regulator for the Notch signalling pathway Su(H) (present in 14.63% of the motifs identified), Adh transcription factor 1 (Adf1) (3.66%), pair-rule gene Runt (Run) (23.17%), Nubbin (Nub) (6.10%), gap genes Giant (Gt) (34.15%), Buttonhead (Btd) (19.51%), and Krüppel (Kr) (3.66%), Smad TF Medea (Med) (14.63%) and POL009.1_DCE_S_II (2.44%) were among the top enriched motifs (p-value < 0.01) in eyespot-specific peaks ( Figure 2, Figure S2: Murugesan and Monteiro, 2023). In the more proximal NES1 wing region, we found 22 motifs that were enriched in the 562 DO peaks of this wing region (Figure S3: Murugesan and Monteiro, 2023). These motifs included binding sites for Pioneer factor Grainy-head (Grh), and TFs involved in the ecdysone signalling pathways such as Ultraspiracle (Usp), Broad (Br), and Hormone receptor-like in 46 (Hr46) (Figure S3: Murugesan and Monteiro, 2023). Similarly, 24 TF binding motifs were enriched in the 785 DO peaks of the more anterior NES2 wing region, with motifs for Hr46, Nubbin (Nub), Odd-skipped (odd), paired (Prd), Toll (Tll) being the most enriched (Figure S4: Murugesan and Monteiro, 2023). 10 TFs motifs were enriched in both NES1 and NES2 DO peaks, relative to eyespots, including Br, Hr46, Pangolin (Pan), and Dorsal (Dl) (Figures S3, S4: Murugesan and Monteiro, 2023).

### Annotation and gene ontology for ATAC peaks

**Figure 3.**
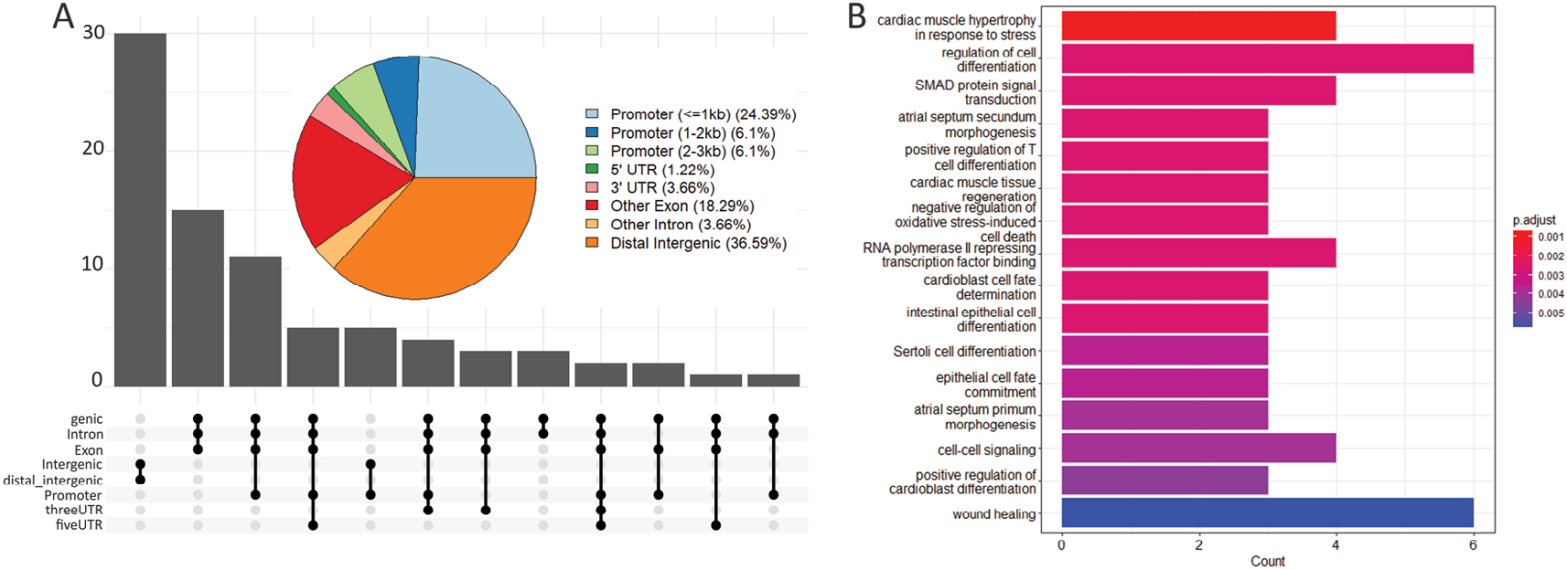
Annotation and GO enrichment for eyespot-specific DO peaks in *B. anynana.* (A) Pie chart and upset plot showing the percentage of the 82 eyespot-specific DO peaks that fall in distinct regions of the genome relative to nearby genes. This includes promoters, distal intergenic regions and UTRs. The maximum number of peaks falls in distal-intergenic and intergenic regions (36.59%). (B) Gene ontology for the 80 genes in the vicinity of the 82 eyespot-specific DO peaks.

In order to identify a potential functional role for the genes in the vicinity of all the identified open chromatin regions, we performed an ontology analysis with those genes. The 166,062 peaks were first annotated for their position in the genome as part of promoters, distal intergenic sequences, or UTRs to nearby genes. More than 50% of the peaks were in distal intergenic regions and around 25% of the regions fell in promoter regions (Figure S5: Murugesan and Monteiro, 2023). Molecular pathways involved in wound-healing, cell differentiation, cell-cell signalling, and SMAD signal transduction were over-represented in the 80 genes found in the vicinity of the 82 eyespot-specific DO peaks ( Figure 3B).

## Discussion

In this work, we examined the chromatin organization of butterfly eyespots and adjacent wing segments to test if the vast gene expression differences found across these different wing regions was also associated with differences in chromatin regulation. Previous transcriptomic experiments showed that ~800 genes were differentially expressed in eyespots relative to adjacent wing sectors, similar in size and location to the ones tested in the current study with ATAC-Seq ( Murugesan *et al.* 2022; Özsu and Monteiro 2017). The large number of DE genes in the eyespot region is believed to be due, in part, to the co-option of a limb gene-regulatory subnetwork to the wing ( Murugesan *et al.* 2022). Here we tested whether the same chromatin profile was present across all three wing segments, regardless of gene expression complexity, or whether pioneer factors, expressed specifically in the eyespots, were producing eyespot-specific open chromatin regions. We found that each of the three sectors of the wing investigated here had a unique chromatin profile, suggesting that DNA from distinct areas of the wing is being opened by sector-specific pioneer factors ( Larson *et al.* 2021; Pihlajamaa *et al.* 2014). This contradicts the findings from McKay and Lieb, (2013) that showed that the only DO peaks between wings, halteres, legs cell homogenates were next to selector genes, such as *Ubx* and *vestigial.*

The large majority of the DE genes in eyespots (~800) had no variation in their chromatin accessibility in eyespot and control tissues. However, there were 82 chromatin regions that were differentially open in eyespots and closed in the other control wing regions. These chromatin regions had an over-representation of DNA motifs recognized by eight transcription factors ( Figure 2) that may represent eyespot-specific pioneer factors or TF that act cooperatively with pioneer factors to regulate eyespot-specific genes. We discuss these genes below.

### Motifs of Notch signalling TF-enriched in eyespot peaks

Eyespots DO peaks were enriched for Su(H) motifs. Su(H) is a transcription cofactor of the Notch signalling pathway ( Baonza and Garcia-Bellido 2000; Bray and Furriols 2001), which can act as a repressor in the absence of Notch signalling ( Bray and Furriols 2001; Larson *et al.* 2021). Notch signalling is activated by the presence of the ligand (Notch) ( Baonza and Garcia-Bellido 2000; Bray and Furriols 2001). This pathway is likely active in eyespots as N shows an eyespot-specific DO peak ( Figure 1C, Data S1) and is expressed in the eyespot centers of *B. anynana* as well as other nymphalid butterfly species ( Oliver *et al.* 2012; Reed and Serfas 2004). N is also one of the four genes whose novel expression in the eyespot centers coincides with the origin of butterfly eyespots at the base of the nymphalids ( Oliver *et al.* 2012; Monteiro 2015). The relative temporal expression of N and Distal-less (Dll) proteins in larval wings, indicated that Notch signalling precedes Dll expression in the eyespot centers ( Reed and Serfas 2004). *Dll* is an essential gene in eyespot development ( Connahs *et al.* 2019), but the role of *N* in eyespot development is not yet known. We propose that eyespot origins might be connected with a region-specific modification of chromatin accessibilities involving Su(H), essential for Notch signalling, in the eyespot regions of the wing.

### Eyespot DO peaks enriched for gap genes motifs

Motifs for the gap gene Btd, which belongs to the family of *Sp* genes, were also enriched in the eyespot-specific open peaks. Ectopic expression of *btd* in dorsal imaginal discs of *Drosophila* (wings, haltere and eyes) led to the formation of ventral structures (antenna and legs) through local activation of segment polarity genes *engrailed* ( *en*) and *wingless* ( *wg*) as well as *decapentaplegic* ( *dpp*) ( Estella *et al.* 2003). Eyespots may be reusing this ancestral limb network, because they also express *en*, *wg* and *dpp* at their centers ( Keys *et al.* 1999; Monteiro *et al.* 2006; Özsu *et al.* 2017; Banerjee *et al.* 2022). As we haven’t observed any differential expression of *btd* in eyespots compared to NSE1 and NSE2 wing tissues ( Murugesan *et al.* 2022; Özsu and Monteiro 2017), further expression analysis with *in situ* probes is required to test the expression of this gene in eyespots at earlier stages of development. Functional analysis should also test its potential role in eyespot development.

Two other gap genes, Kr and Gt, showed enrichment for eyespot-specific open chromatin regions. Kr acts as a downstream target gene in Notch-mediated cell differentiation in Malpighian tubules (MT) in *Drosophila* ( Hoch and Jäckle 1998). Homologs of Kr in humans are called Krüppel-like factors (KLFs), and are involved in various cellular mechanisms along with Sp1-like proteins. KLF4 in humans is one of the pioneer factors that regulates cellular division, proliferation and differentiation ( Borisova *et al.* 2022; Farrugia *et al.* 2016; Kaczynski, Cook, and Urrutia 2003). KLF4 along with Sox2, Oct4 and Nanog are essential for maintaining stem cell properties and their activation in somatic cells enables induced pluripotency ( Liu *et al.* 2020). The enrichment of Kr in eyespot-specific peaks suggests that Kr could likely act as a potential pioneer factor under the influence of Notch signalling to open the eyespot peaks.

Gt is a gap gene that when disrupted shows broad regions of the body missing as well as segmentation defects ( Bucher and Klingler 2004; Brent *et al.* 2007). Gt and Kr are expressed in a complementary non-overlapping manner in early *Drosophila* embryos, mutually repressing each other ( Kraut and Levine 1991). It would be interesting to investigate the expression pattern of both genes in and around the eyespots in future.

In addition, TFs Nub and Med motifs were also enriched in eyespot-specific open peaks. Nub belongs to a POU-family of TFs. It is expressed in early wing primordium and is required for proximal-distal patterning of wings in *Drosophila* ( Ng, Diaz-benjumea, and Cohen 1995; Zirin and Mann 2007). Med is a genetic modifier of Dpp signalling. It regulates wing patterning along the anterior-posterior axis through binding with the selector gene scalloped (sd) in *Drosophila* ( Guss *et al.* 2001). The function of these TFs in eyespot development should be investigated in future.

Given the importance of ecdysone signalling and the presence of its receptor Ecdysone receptor (EcR) in eyespot centers, we looked for enrichment of EcR and other ecdysone signalling transcription factors that may play important roles in shaping the chromatin dynamics in the eyespot centers, compared to control tissues. However, we didn’t see any motif enrichment for EcR or other ecdysone TFs. For a long time, EcR has been thought to be a pioneer factor responding to ecdysone signalling and changing chromatin architecture ( Uyehara and McKay 2019). Recent research in *Drosophila* wings and salivary glands *,* however, showed that EcR binds opportunistically to chromatin previously opened by tissue-specific pioneer factors, in particular Forkhead and Grainyhead ( Uyehara *et al.* 2022). Also, in *Junonia coenia* butterflies, Spineless binding sites were enriched in chromatin peaks that were dynamically opening during wing metamorphosis, whereas EcR binding sites were only enriched in persistently open sites throughout development ( van der Burg *et al.* 2019). These data point to EcR not being a pioneer factor for wing development. However, this needs to be further tested, as here we picked a single developmental stage, 3 h after pupation, before a large surge in 20E titers. Analysis of chromatin peaks right after this surge, not examined here, could have distinct motif enrichments, including those for EcR.

### Ecdysone signalling gene showing eyespot specific DO peaks

Hormone receptor-like 39 (Hr39) also showed eyespot-specific chromatin accessibilities (Figure S1: Murugesan and Monteiro, 2023). Hr39 is an orphan nuclear hormone receptor considered as a master regulator with a conserved role in female reproductive glands and secretive tissues in animals ( Cattenoz *et al.* 2016; Praggastis *et al.* 2021). Hr39 is also a downstream canonical ecdysone response gene likely regulated through the EcR in wings ( Uyehara and McKay 2019). Many butterfly eyespots express EcR at their center, and in *B. anynana* the expression of EcR in eyespot centers is tightly controlled by the ecdysone signalling pulse ( Monteiro *et al.* 2015). The evolution of eyespots in nymphalid butterflies is also coincident with the novel expression of EcR in the eyespot centers ( Bhardwaj *et al.* 2020). The enrichment of Hr39 binding sites in the open chromatin peaks specific to eyespot tissue could have facilitated ecdysone signalling in the eyespot centers and eyespot origins.

In summary, eyespot regions of the wing had areas of open chromatin that had an enrichment of DNA motifs recognized by a potential pioneer factor like Kr, as well as several other TFs. Some of these TFs have previously been suggested to be involved in eyespot development. Others should be examined in the future, including Kr.

### Eyespot-specific peaks map to genes involved in wound healing

To test if the genes next to the eyespot-specific open peaks were involved in any of the previously proposed signalling or molecular pathways involved in eyespot development, we performed gene ontology analysis. We found that wound healing is one of the significantly enriched gene ontologies along with SMAD signal transduction, cell-cell signalling and cell differentiation ( Figure 3).

Previous work on eyespot DE genes using RNA-Seq highlighted many genes involved in wound-healing mechanisms ( Özsu and Monteiro 2017), including Notch signalling ( Vizcaya-Molina *et al.* 2018). Piercing pupal wings produces ectopic eyespots and activates genes involved in eyespot development in the wounded region ( Monteiro *et al.* 2006), suggesting that eyespot and wound healing GRNs share common genes and regulatory elements. In our previous work, we identified two ATAC-Seq peaks next to *Distal-less* and *spalt* that were open in eyespots, whole wings, as well as wounded wings ( Murugesan *et al.* 2022). The extent that additional genes and regulatory elements are shared between eyespots and wound healing needs to be further explored to understand how these two GRNs are related.

SMAD signal transduction was also enriched in our gene ontology analysis. SMAD proteins are the intracellular mediators of both Dpp and Wnt signalling ( Wrana and Attisano 2000), and both Smad2 and Smad6 are differentially expressed in the eyespot centers ( Murugesan *et al.* 2022). Furthermore, both Dpp and Wingless are potential morphogens involved in eyespot development, and CRISPR knockout of both genes led to the loss of eyespots ( Banerjee *et al.* 2022).

We propose that co-option of pre-existing GRNs, such as the limb GRN ( Murugesan *et al.* 2022) and/or the wound healing GRN ( Özsu and Monteiro 2017) to the novel location where eyespots develop was potentially mediated via the generation of a novel eyespot-specific open chromatin region next to a top-regulator of the network. This would allow that top regulator to have a novel activity in a novel domain of the body of the butterfly. Any of the eighty genes with DO chromatin identified in this study might be such a top regulator. Furthermore, we propose that Kr, the only confirmed pioneer factor whose binding sites were enriched in the eyespot-specific open chromatin, is a good candidate for mediating these eyespot-specific patterns of open chromatin.

Overall, our study shows that eyespots show differential chromatin accessibility compared to two other nearby wing regions. Eyespot-specific accessibility peaks are enriched for motifs and gene ontology corresponding to many of the genes and pathways previously proposed to be involved in eyespot development. This clearly demonstrates that chromatin accessibility varies across the wing, and with specific wing patterns in *B. anynana* butterflies.

### Limitations of the study

The main limitation of this study lies in the tissue collection method adopted to distinguish eyespot from non-eyespot cells. Though we were able to capture differences in chromatin accessibilities between the different sections of the wings, dissections of eyespot tissues contain non-eyespot cells which might hinder the precision in identifying the ATAC peaks corresponding to eyespot cells alone. One way to overcome this limitation in future is to use single-cell sequencing approaches, and eyespot-specific marker genes, to study ATAC-peaks only on those marker-expressing candidate cells.

## Methods

*Bicyclus anynana* larvae and adults were reared in 27°C and 60% humidity inside a climate room with 12:12 h of dark and light cycle. Adults were fed with bananas and larvae were fed on young corn plants.

### ATAC-Seq Library preparation

ATAC-Seq was performed for the tissues as described in Murugesan *et al.* (2022). Two replicates were made for the eyespot-specific sector and three replicates for each control sector. Eyespot dataset was taken from Murugesan *et al.* (2022). The control tissues NES1 and NES2 were from the same forewings as eyespot tissues dissected around 3 h to 6 h after pupation. We dissected as many tissues as possible within 15 minutes, and then snap-froze them using liquid nitrogen. Tissues were stored at -80°C before nuclei extraction. We pooled 50 wing bits from the same wing area to produce a single biological replicate, which contained approximately 80,000 nuclei. Prepared libraries were sequenced using Novoseq 6000, with an average read depth of 30 million reads per library and 2×50 bp paired-end at Novogene, Singapore. We also used 3 h pupal whole wing data from Murugesan *et al.* (2022) as a control dataset for the peaks identified from micro dissections.

### ATAC-Seq analysis

ATAC-Seq analysis was carried out as described in Murugesan *et al.* (2022) with few modifications. Reads obtained from the sequencing were processed using bbduk scripts from bbmap tools ( Bushnell 2014) to remove any adapters. Reads were mapped to *Bicyclus anynana* genome, version BaGv2 ( Murugesan *et al.* 2022) using bowtie. Reads mapped to the mitochondrial genome and marked for duplicates were removed using samtools idxstats and GATK MarkDuplicates respectively. Peak calling was done using Fseq ( Boyle *et al.* 2008). Consensus peaks for all samples were obtained using bedtools intersect, followed by bedtools merge with peaks within 50 bp distance from each other resulting in a total of 166,062 peaks. The consensus peaks were used for all downstream analyses. FRiP score was used to measure the fraction of reads mapped to the genome falling into peaks. Our ATAC-Seq data showed a median FRiP score of 0.41 which is greater than the ENCODE standard (>0.3) ( Table 1). Deeptools ( Ramírez *et al.* 2014) were used to measure the correlation between replicates and quality of the ATAC-Seq data. A read count matrix was produced corresponding to the consensus peaks for the libraries using Featurecount from Subread tools ( Liao, Smyth, and Shi 2014). Differential peak analysis was performed using DESeq2 ( Love, Huber, and Anders 2014) and the peaks were considered differentially accessible with a p-value < 0.05. Peaks were called differentially open (DO) if their logFC > 0 and p-value ≤ 0.05. Quality check for the ATAC data was done using Deeptools (Figure S6: Murugesan and Monteiro, 2023).

**Table 1.** ATAC-Seq reads. Read depth and FRiP score.

Samples	Reads mapped to genome	Reads mapped to peaks	FRiP score
eyespot1	11795797	5530410	0.46884581
eyespot2	12147031	5389137	0.443658784
NES1_1	14805085	6125405	0.413736564
NES1_2	11307756	4781473	0.422848972
NES1_3	18576805	7648763	0.411737271
NES2_1	12595930	4937158	0.391964547
NES2_2	10935418	4329065	0.395875585
NES2_3	11602454	4805120	0.414146869

### ATAC-peaks annotation

Consensus ATAC peaks and subset of peaks obtained from the differential peaks were all annotated using the R package ChiPseeker ( Bushnell 2014). The gene annotation gff file for BaGv2 genome version was loaded into the pipeline using GenomicFeatures ( Lawrence *et al.* 2013) library and was used to locate the position of peaks in the genome with respect to the gene structures. Promoters were defined as peaks 3000 bp upstream and downstream of the gene’s transcription start site. The distal-intergenic peaks are mapped to genes which are within 300 Kb from the transcription start site (Data S1)

### Gene ontology for differential ATAC peaks

Genes corresponding to the differential peaks were obtained from the ChiPseeker annotation. Gene ontology was carried out as described in Murugesan *et al.* (2022). Gene ontology for the BaGv2 genome was obtained from Interproscan ( Jones *et al.* 2014). ClusterProfiler package ( Wu *et al.* 2021) in R was used to identify the enriched gene ontology for the given gene lists.

### Motif enrichment analysis

Homer *findMotifsGenome.pl* ( Heinz *et al.* 2010) was used to identify enriched motifs in differentially accessible regions identified from DESEq2. Bed files for the peaks of interest were produced. BaGv2 genome was incorporated into Homer to run locally. Transcription factor binding motifs were searched just within the peaks’ exact sizes, with GC normalization for the background sequences and in insect databases in Homer.

## Authors contribution

SNM and AM designed the study. SNM carried out the experiment and analysed the data. SNM and AM wrote the manuscript. All authors read and approve the manuscript.

## Data Availability

NCBI BioProject: *Bicyclus anynana* (squinting bush brown). Accession number: PRJNA685019; ATAC-Seq reads for eyespot and control tissues are submitted in NCBI under Bioproject, https://identifiers.org/bioproject:PRJNA685019 ( Murugesan *et al*. 2023). Biosamples: SAMN17214324, SAMN17214325, SAMN33923641, SAMN33923642, SAMN33923643, SAMN33923644, SAMN33923645, SAMN33923646. DRYAD: Data for: Butterfly eyespots exhibit unique patterns of open chromatin, https://doi.org/10.5061/dryad.q573n5tp9 ( Murugesan and Monteiro 2023). This project contains the following extended data:
- Data for: Butterfly eyespots exhibit unique patterns of open chromatin - Figure S1 - Figure S2 - Figure S3 - Figure S4 - Figure S5 - Figure S6 Data are available under the terms of the Creative Commons Zero “No rights reserved” data waiver (CC0 1.0 Public domain dedication).
