# Supplementary material for: Butterfly eyespots exhibit unique patterns of open chromatin
Source: F1000Res. 2023 Oct 31;12:1428. [Version 1] doi: 10.12688/f1000research.133789.1 (PMC11109672; doi:10.12688/f1000research.133789.1)
Supplement: Peer review history

# Reviewer report: Reviewer response for version 1

**DOI:** 10.5256/f1000research.146802.r232273

**Published:** 2024-05-11

**Authors:** Craig Brunetti

**Affiliations:** 1 Trent University, ON, Canada

Previous studies on insect whole-body parts have demonstrated little variation in chromatin accessibility.  More recently, studies have shown that in fact variation may exist if smaller regions or single cells are tested.  The rationale is that the average of chromatin accessibility over a body part may mask variations within subsets of the tissue.  The manuscript “Butterfly eyespots exhibit unique patterns of open chromatin” explores chromatin accessibility in various pattern elements on the wings of *Bicyclus anynana*. ATAC-Seq was performed on 3hr pupal wing discs from either the eyespot or adjacent non-eyespot tissue.  The authors demonstrated that there were distinct open chromatin regions in the eyespot-specific tissue versus control tissue.  Areas of open chromatin corresponded to pathways previously implicated in eyespot formation.  These data suggest that there is variation in open chromatin across tissues and provides further insight into the patterning of wings of butterflies.

 Some comments related to the manuscript:

1. In the 5 ^th^ paragraph of the Introduction, second sentence, change “control regions next door” to “adjacent control regions”
2. Figure 3B shows a large % of the genes in the eyespot DO set are involved in wound healing.  There should be a mention in the discussion about why this isn’t the result of wound healing induced during surgical removal of the tissue.  Highlighting the fact that the tissue is removed quickly and fast frozen (within 15 minutes) along with the fact that this is differential expression and induction of wound healing would happen in all tissue (eyespot and control).

Is the work clearly and accurately presented and does it cite the current literature?

Yes

If applicable, is the statistical analysis and its interpretation appropriate?

Yes

Are all the source data underlying the results available to ensure full reproducibility?

Yes

Is the study design appropriate and is the work technically sound?

Yes

Are the conclusions drawn adequately supported by the results?

Yes

Are sufficient details of methods and analysis provided to allow replication by others?

Yes

Reviewer Expertise:

virology, immunology, butterfly wing patterning.

I confirm that I have read this submission and believe that I have an appropriate level of expertise to confirm that it is of an acceptable scientific standard.

# Reviewer report: Reviewer response for version 1

**DOI:** 10.5256/f1000research.146802.r267378

**Published:** 2024-04-19

**Authors:** Richard ffrench-Constant

**Affiliations:** 1 University of Exeter, Cornwall, Penryn, UK

This is a really nice paper that begins to fill the gap between gene and phenotype in butterfly wing pattern formation. By carefully staging and then carefully dissecting developing eyespot tissue the authors have shown how chromatin structure differs with wing scale development. This now allows us to identify potential transcription factors in an unbiased manner (in other words not simply looking for those for which we already have probes or antibodies). This sort of approach should pay dividends if used across other butterfly wing pattern elements. Congratulations to the authors.

Is the work clearly and accurately presented and does it cite the current literature?

Yes

If applicable, is the statistical analysis and its interpretation appropriate?

Yes

Are all the source data underlying the results available to ensure full reproducibility?

Yes

Is the study design appropriate and is the work technically sound?

Yes

Are the conclusions drawn adequately supported by the results?

Yes

Are sufficient details of methods and analysis provided to allow replication by others?

Yes

Reviewer Expertise:

butterfly wing pattern formation

I confirm that I have read this submission and believe that I have an appropriate level of expertise to confirm that it is of an acceptable scientific standard.
